# Supplementary material for: Molecular Architecture of Cryptococcus Cell Walls Reveals Species-Specific Chitosan-Dependent Remodeling
Source: bioRxiv. 2026 Apr 15:2026.04.14.718415. Preprint. [Version 1] doi: 10.64898/2026.04.14.718415 (PMC13104905; doi:10.64898/2026.04.14.718415)
Supplement: Supplement 1 [file media-1.pdf]

Supplementary Information

Molecular Architecture of *Cryptococcus* Cell Walls Reveals Species-Specific Chitosan-Dependent Remodeling

Ankur Ankur[1#], Rajendra Upadhya[2*#], Mahsa Doosti[1], Davis Ferreira[3], Li Xie[1], Ivan Hung[4], Jennifer K. Lodge[2*], Tuo Wang[1*]

[1]Department of Chemistry, Michigan State University, East Lansing, MI, USA

[2]Department of Molecular Genetics and Microbiology, Duke University School of Medicine, Durham, NC, United States

[3]Department of Pathology, Duke University School of Medicine, Durham, NC, United States

[4]National High Magnetic Field Laboratory, Florida State University, Tallahassee, FL, USA

[#] These authors contributed equally

[*] Correspondence: rajendra.upadhya@duke.edu; jennifer.lodge@duke.edu; wangtuo1@msu.edu

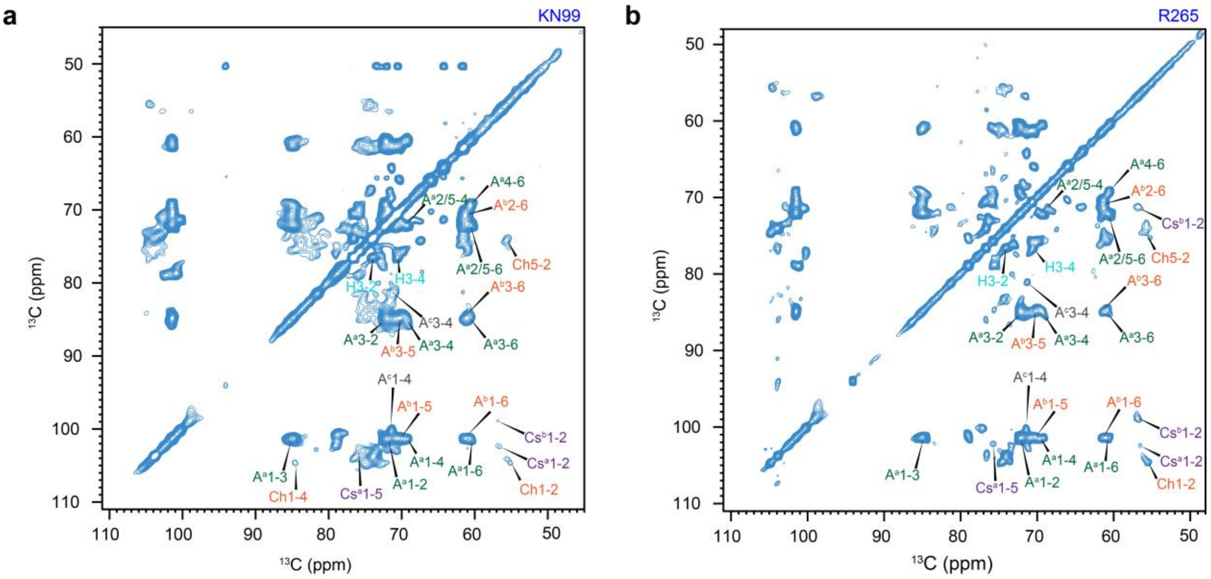

Supplementary Figure 1. Resonance assignment of rigid glucans in *C. neoformans* and *C. gattii* cell wall. CP-based 2D ^{13}C-^{13}C correlation spectrum measured with 53 ms CORD mixing for **(a)** KN99, and **(b)** R265.

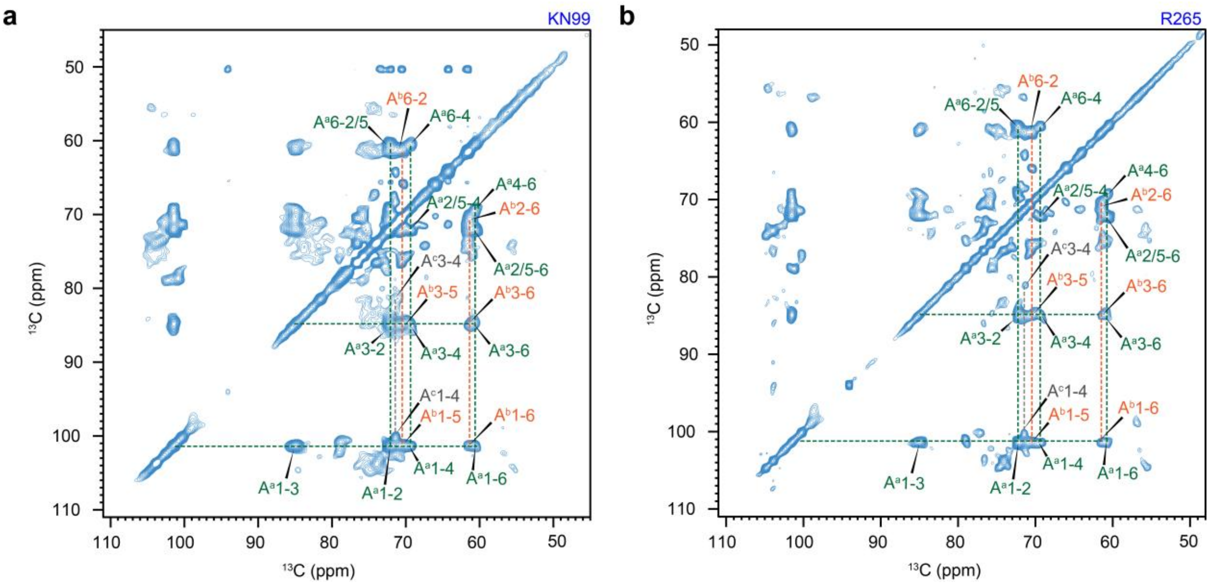

Supplementary Figure 2. Resonance assignment of α-1,3-glucan in *C. neoformans* and *C. gattii* cell wall. CP-based 2D ^{13}C-^{13}C correlation spectrum measured with 53 ms CORD mixing. **(a)** KN99, and **(b)** R265. Each peak is annotated with the abbreviation of the carbohydrate name, the subtype (in superscript), and the carbon number. For instance, A^c1 represents the carbon 1 of type-c α-1,3-glucan.

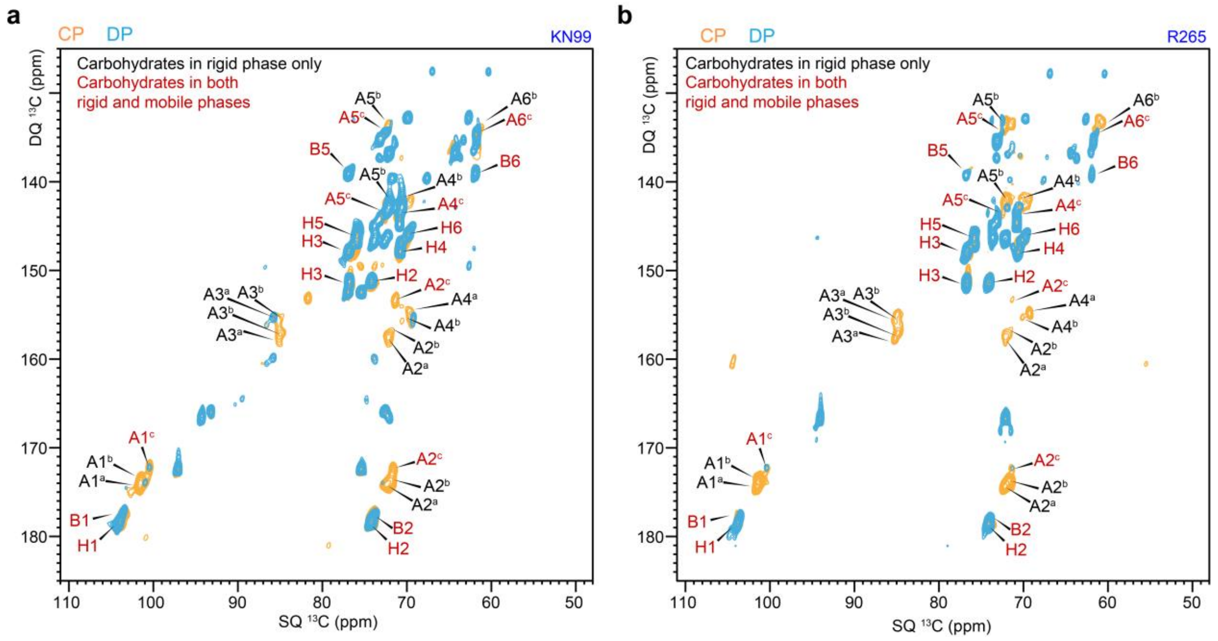

Supplementary Figure 3. Distribution of α-1,3-glucan, and β-glucan in rigid and mobile domains. Overlay of 2D refocused J-INADEQUATE spectra measured with CP (orange) and DP (cyan) for **(a)** *C. neoformans* KN99 and **(b)** *C. gattii* R265 samples. The carbohydrates observed only in the CP-based spectra are rigid and are marked in black. The carbohydrates observed in both CP-INADEQUATE and CP-INADEQUATE spectra are marked in red: these carbohydrates have two-modal distribution in rigid and mobile phases. β-1,6-glucan, β-1,3-glucan, and types-c α-1,3-glucan were observed in both domains for both samples.

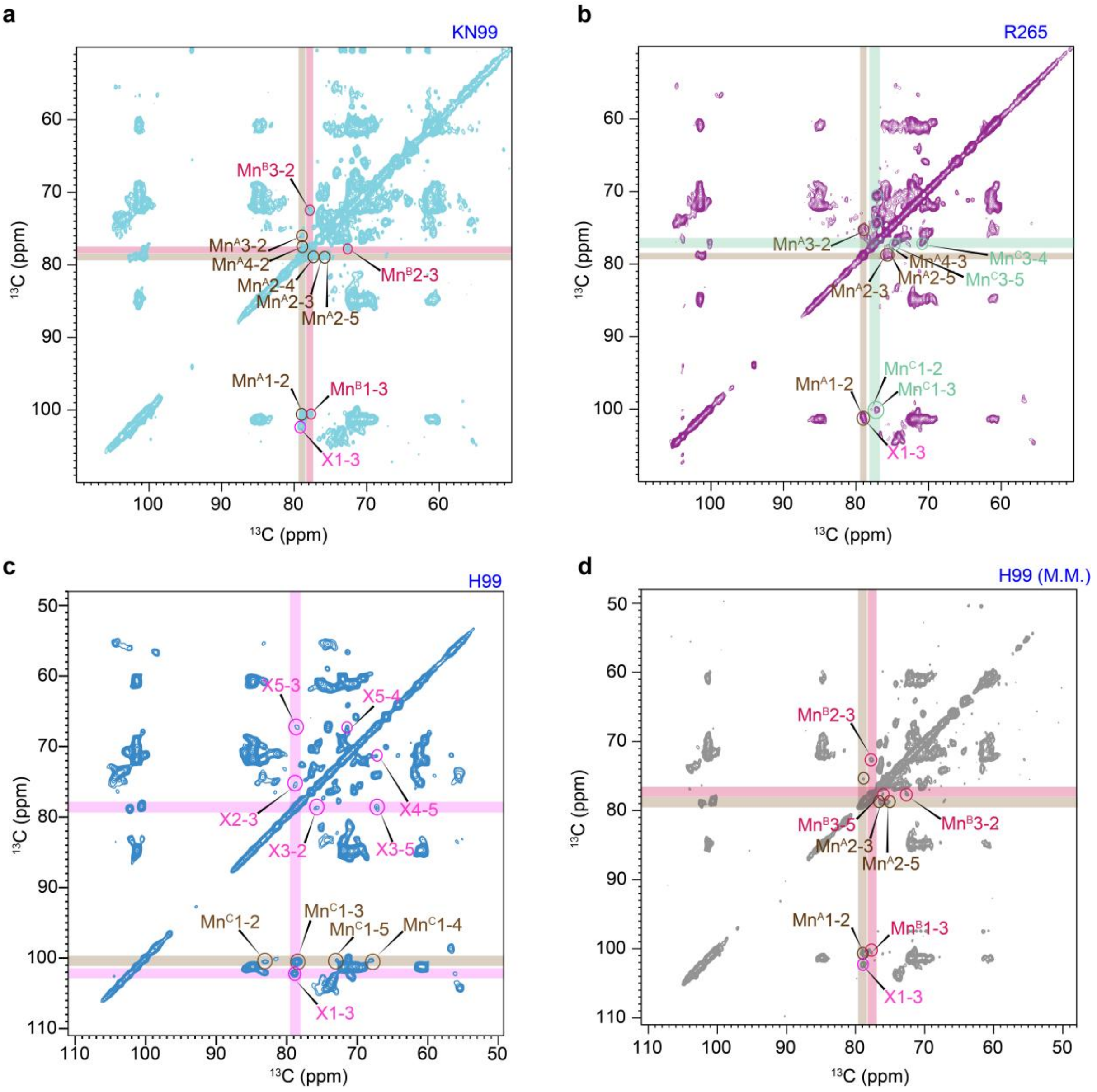

Supplementary Figure 4. Resonance assignment of capsular molecules in *Cryptococcus*. CP-based 2D ^{13}C-^{13}C correlation spectrum measured with 53 ms CORD mixing for different samples of *cryptococcus*, highlighting xylose and different types of mannose signals arising from GXM unit of capsules. **(a)** *C. neoformans* KN99, **(b)** *C. gattii* R265, **(c)** *C. neoformans* H99, and **(d)** *C. neoformans* H99 grown in minimal media.

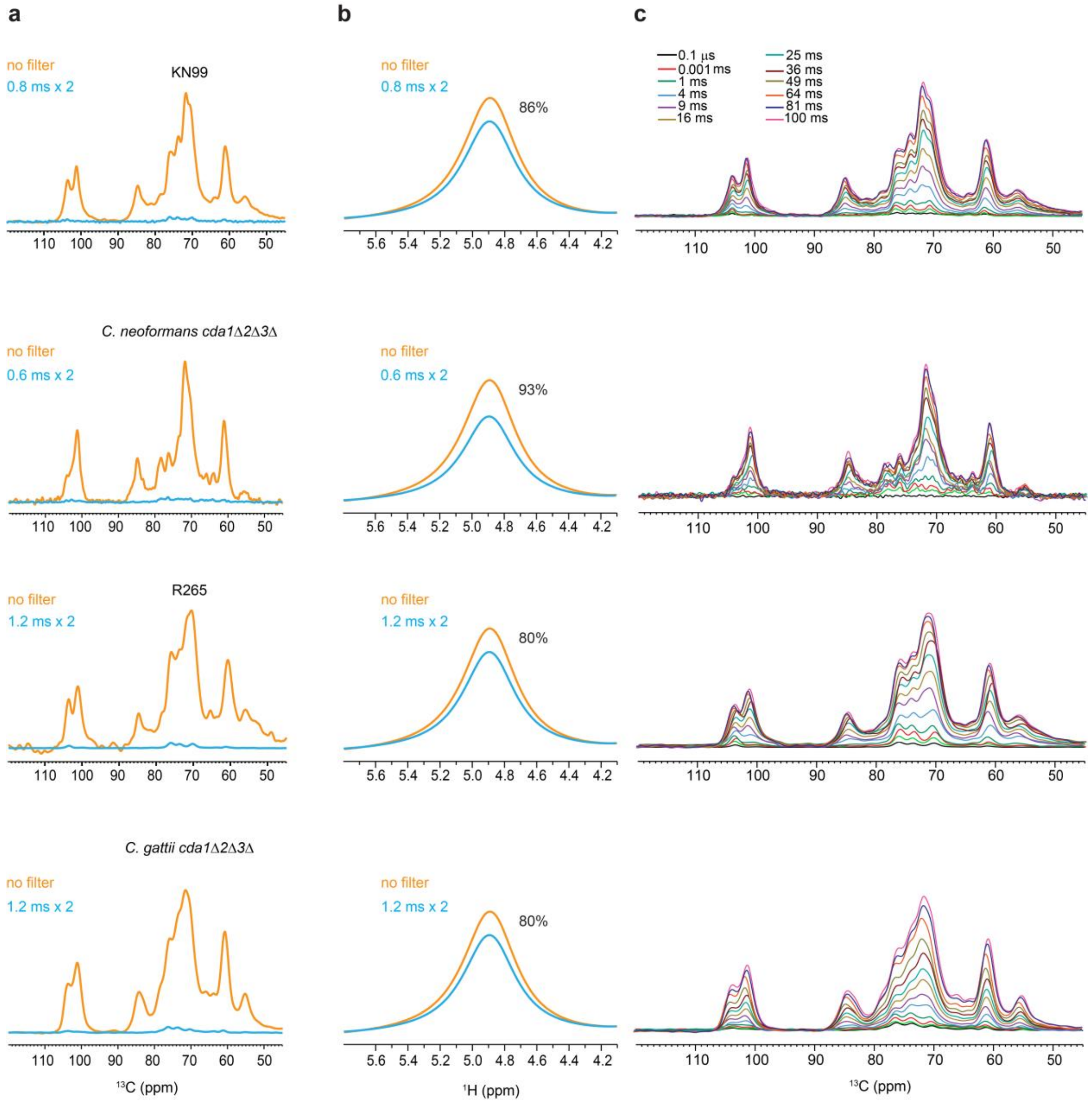

Supplementary Figure 5. Water-edited experiment setup for inspecting carbohydrate hydration. **(a)** ^{1}H-T$_2$ filtered (blue) and control (orange) ^{13}C spectra are shown for four strains. From the top to bottom: KN99, *C. neoformans cda1Δ2Δ3Δ*, R265, and *C. gattii cda1Δ2Δ3Δ*. No spin diffusion was applied. Approximately 85% of carbohydrate ^{13}C signals were removed by the T$_2$ filter. **(b)** ^{1}H-T$_2$ filtered (blue) and control (orange) ^{1}H NMR spectra, with 80% and 93% of water signal retained for each strain, after the ^{1}H T$_2$ filter. From the top to bottom: KN99, *C. neoformans cda1Δ2Δ3Δ*, R265, and *C. gattii cda1Δ2Δ3Δ*. **(c)** 1D water-edited ^{13}C spectra with different ^{1}H mixing times. From the top to bottom: KN99, *C. neoformans cda1Δ2Δ3Δ*, R265, and *C. gattii cda1Δ2Δ3Δ*. All spectra were measured on 400 MHz spectrometer at 15 kHz MAS at 280 K.

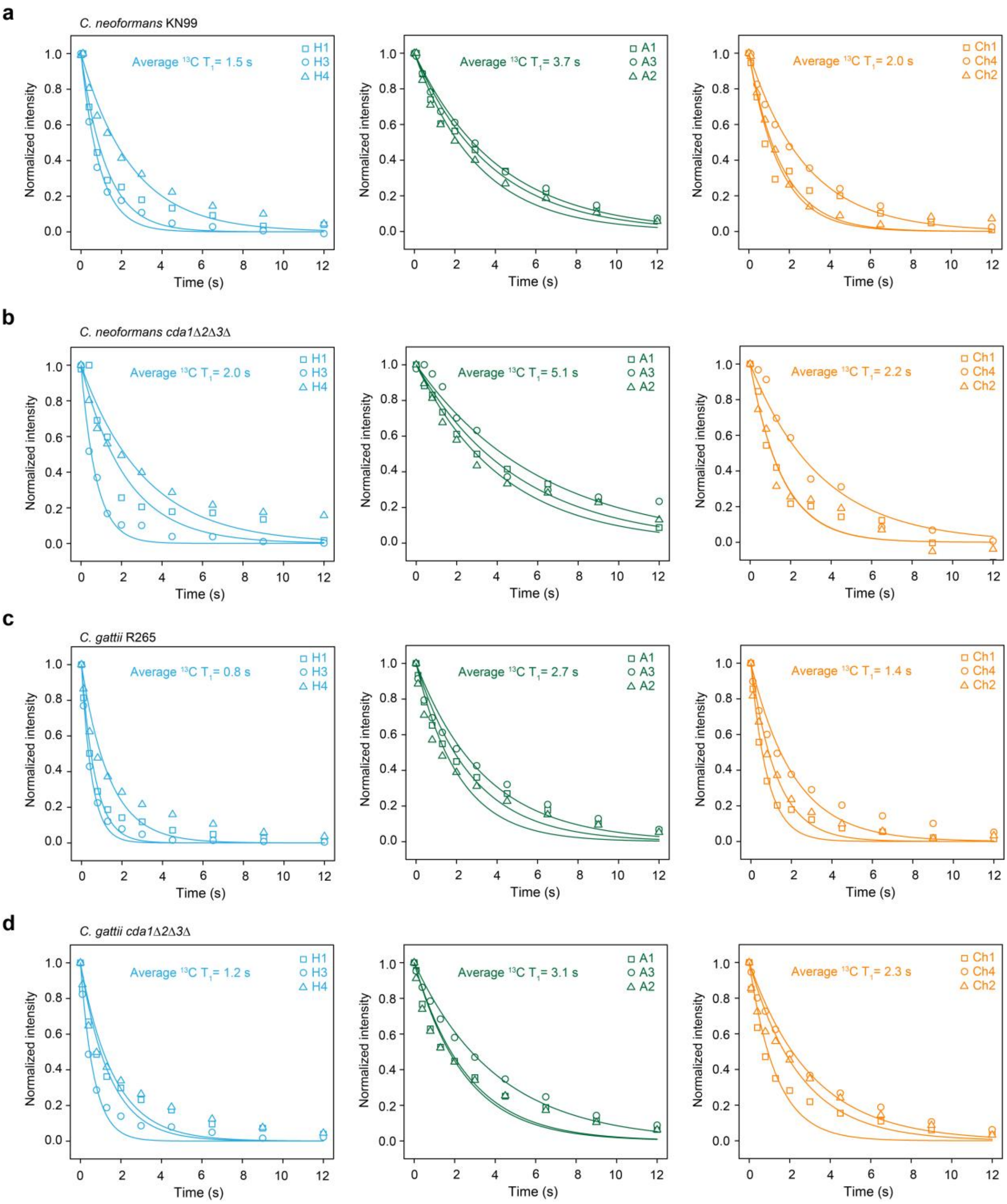

Supplementary Figure 6. ^{13}C-T_1 relaxation of polysaccharides in *C. neoformans* and *C. gattii*. ^{13}C-T_1 measured with Torchia CP for **(a)** KN99 **(b)** *C. neoformans cda1Δ2Δ3Δ* **(c)** R265 and **(d)** *C. gattii cda1Δ2Δ3Δ* samples. The data are separately presented for β-1,6-glucan (light blue), α-1,3-glucan (green), and chitin (orange). The acquired data were fitted to a single exponential decay equation. Different symbols and color codes are used to represent different carbons in these polysaccharides.

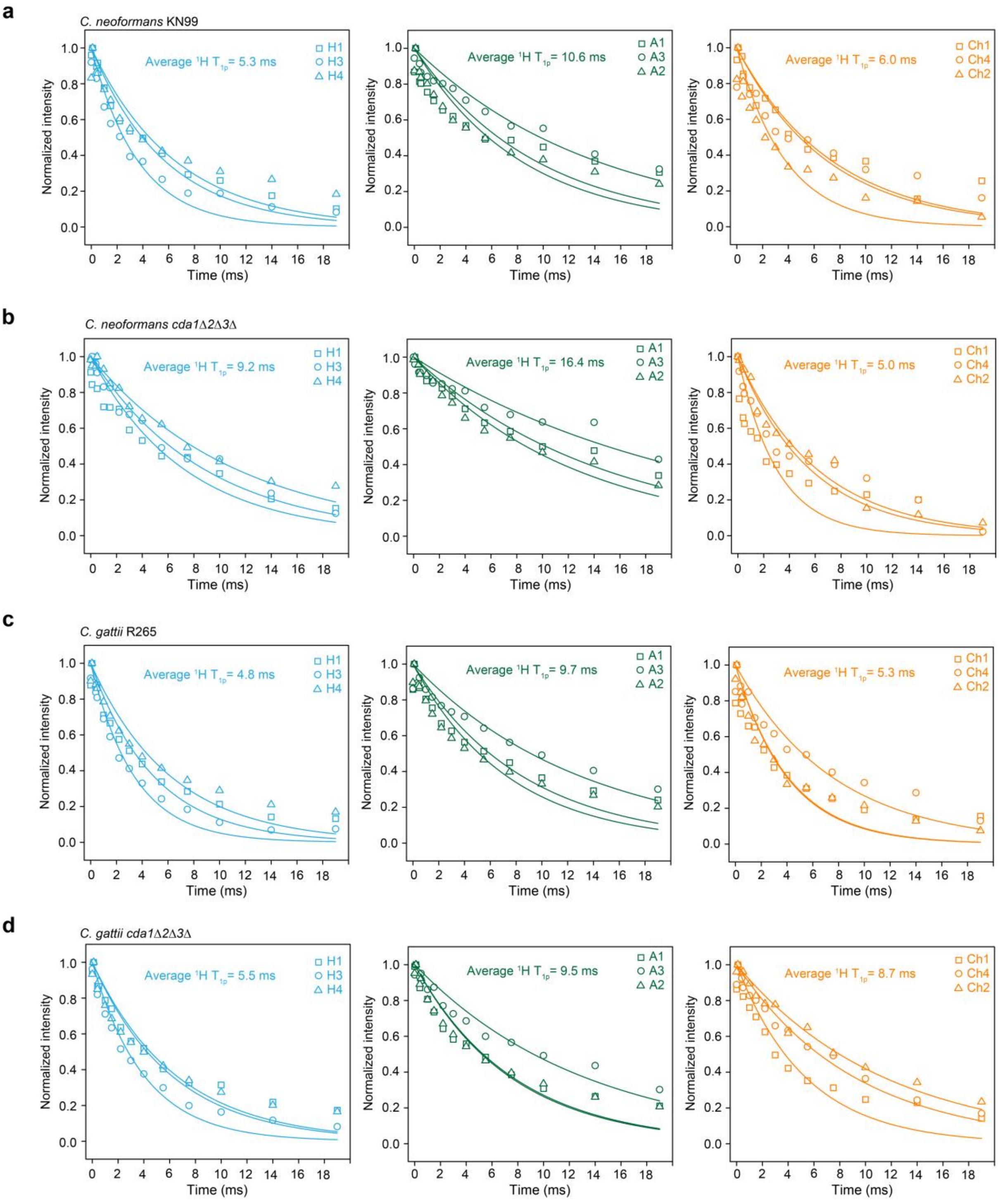

Supplementary Figure 7. ^{1}H-T$_{1\rho}$ relaxation of polysaccharides in *C. neoformans* and *C. gattii*. ^{1}H-T$_{1\rho}$ measured with Torchia CP for **(a)** KN99 **(b)** *C. neoformans cda1Δ2Δ3Δ* **(c)** R265 and **(d)** *C. gattii cda1Δ2Δ3Δ* samples. The data are separately presented for β-1,6-glucan (light blue), α-1,3-glucan (green), and chitin (orange). The acquired data were fitted to a single exponential decay equation. Different symbols and color codes are used to represent different carbons in these polysaccharides.

Supplementary Table 1. ^{13}C chemical shifts of *C. neoformans* cell wall and capsular polysaccharides in cell walls from ^{13}C-based experiments. The referencing scale is TMS scale. All chemical shifts are from room-temperature experiments.

Carbohydrates	form	C1	C2	C3	C4	C5	C6	Reference
Rigid molecules								
α-1,3-glucan (A)	a	101.6	72.4	85.5	69.4	71.2	61.4	Chakraborty *et al.* 2021[1]
	b	101.4	71.8	85.0	70.5	72.2	60.7	
	c	100.4	71.5	81.9	71.1	72.6	61.6	
β-1,6-glucan (H)		103.8	74.2	76.8	70.8	75.8	69.8	Lowman *et al.* 2011[2]
Chitin		104.3	55.3	73.7	83.7	75.7	61.1	Kang *et al.* 2018[3] Fernando *et al.* 2021[4]
Chitosan	a	102.1	56.5	73.3	83.4	75.6	60.3	
	b	98.6	56.9	70.9	/	/	/	
Mannan	Mn^A	100.8	78.8	76.1	77.1	75.4	/	Bacon *et al.* 1996[5] Hargett *et al.* 2024[6] Previato *et al.* 2017[7] Ankur *et al.* 2025[8]
	Mn^B	100.6	72.5	77.8	/	76.0	/	
	Mn^C	100.1	77.8	77.0	70.7	74.5	/	
Xylose		102.2	75.8	78.8	71.3	67.1	n.a.	
Mobile molecules								
β-1,6-glucan (H)		103.8	74.2	76.8	70.8	75.8	69.8	Lowman *et al.* 2011[2]
β-1,3-glucan (B)	a	103.6	73.7	86.5	69.3	76.8	62.0	Chakraborty *et al.* 2021[1] Shim *et al.* 2007[9] Fairweather *et al.* 2009[10] Saito *et al.* 1979[11]
	b	103.6	73.7	85.8	69.3	76.8	62.0	
α-1,3-glucan (A)	a	/	/	/	/	/	/	
	b	/	/	/	/	/	/	
	c	100.4	71.5	81.9	71.1	72.6	61.6	
α-1,2-Mannan ($Mn^{1,2}$)		100.7	78.7	71.3	67.2	74.0	61.7	Chakraborty *et al.* 2021[1]
α-1,6-Mannan ($Mn^{1,6}$)		102.9	70.8	/	/	71.3	64.0	
Xylose (X)		102.7	73.2	76.2	70.2	66.0	n.a.	Previato *et al.* 2017[7] Ankur *et al.* 2025[8]

Supplementary Table 2. The molar composition of rigid polysaccharides. The numbers are estimated using integrals (volume) of cross peaks in 2D ^{13}C-^{13}C 53 ms CORD spectra. The average integrals of cross-peaks of each polysaccharide are shown. Error bars are standard errors.

	Polysaccharide								
Strain	α-1,3-glucan			β-1,6-glucan	Chitin	Chitosan		Mannan	Xylose
	a	b	c			a	b		
C. neoformans KN99	36±12	26±3	9±3	15±4	3±0.4	3±0.4	2±0.4	2±1	4±0.6
C. neoformans cda1Δ2Δ3Δ	42±12	28±6	6±2	7±2	7±2	/	/	7±4	3±1
C. gattii R265	24±7	17±3	6±2	25±7	6±1	3±0.3	4±0.4	9±2	6±1
C. gattii cda1Δ2Δ3Δ	24±8	16±2	/	23±2	13±3	/	/	10±4	14±2

The area of the following well-resolved cross peaks 53 ms CORD spectra are used:
α-1,3 (a): the average of C1-C2/3/4 and C3-C2/4.
α-1,3 (b): the average of C1-C2/4, C3-2/4.
α-1,3 (c): the average of C1-C2/4, C3-2/4.
β-1,6: the average of C3-C2/4, C5-C4/6.
Chitin: the average of C1-2/5/6, C4-C2, C5-C2.
Chitosan (a): the average of C1-2/3/5, C3/5-C2.
Chitosan (b): the average of C1-2, C3-C2.
Mannan: the average of C1-C2, C2-C5, C5-4.
Xylose: the average of C1-C3, C2-3.

Supplementary Table 3. The molar composition of mobile polysaccharides. The numbers are estimated using integrals (volume) of cross peaks in 2D ^{13}C-^{13}C refocused DP-J INADEQUATE spectra. The average integrals of cross-peaks of each polysaccharide are shown. Error bars are standard errors of the peak integrals. $Mn^{1,2}$ signals in R265 cells have low intensity.

Polysaccharide						
Strains	β-1,6-glucan	β-1,3-glucan	α-1,3-glucan (c)	Mannan ($Mn^{1,2}$)	Mannan ($Mn^{1,6}$)	Xylose
C. neoformans KN99	79±13	8±2	2±1	/	11±2	/
C. neoformans cda1Δ2Δ3Δ	53±7	/	7±1	14±2	12±2	14±2
C. gattii R265	83±14	4±1	5±1	/	8±3	/
C. gattii cda1Δ2Δ3Δ	74±12	8±3	/	/	12±3	6±1

The area of the following well-resolved cross peaks refocused DP-J INADEQUATE spectra are used:
β-1,6: the average of C1, C2, C3, C4, C5, and C6.
β-1,3; the average of C1, C2, C3, C4, C5, and C6.
α-1,3-glucan: the average of C1, C2, and C3.
$Mannan^{1,2}$: the average of C1, C2, C3, C4, C5 and C6.
$Mannan^{1,6}$: the average of C1, C2, C3, C4, C5 and C6.
Xylose: the average of C1, C2, C3, C4, C5 and C6.

Supplementary Table 4. Water-edited intensities of polysaccharides. Intensity ratios are obtained by comparing the peak intensities in water-edited and control spectra. The average values for each molecule in each sample are highlighted. Error bars are s.d. propagated from NMR signal-to-noise ratios.

Polysaccharide	Cross-peak	*C. neoformans*		*C. gattii*	
		KN99	*cda1Δ2Δ3Δ*	R265	*cda1Δ2Δ3Δ*
α-1,3-glucan (A^a)	A1-1	0.49±0.06	0.51±0.03	0.33±0.04	0.25±0.02
	A1-3	0.54±0.08	0.55±0.08	0.29±0.01	0.36±0.06
	A1-2/5	0.56±0.02	0.47±0.04	0.46±0.05	0.28±0.03
	A1-A4	0.52±0.03	0.53±0.06	0.28±0.01	0.27±0.05
	A3-A1	0.32±0.08	0.26±0.05	0.40±0.02	0.34±0.07
	A3-A3	0.62±0.05	0.36±0.06	0.40±0.08	0.43±0.05
	A3-2/5	0.47±0.06	0.59±0.06	0.44±0.07	0.28±0.04
	A3-4	0.36±0.06	0.58±0.06	0.53±0.02	0.21±0.03
	A2/5-1	0.49±0.04	0.50±0.01	0.44±0.04	0.20±0.03
	A2/5-3	0.54±0.07	0.40±0.01	0.45±0.08	0.22±0.04
	A2/5-2/5	0.58±0.01	0.53±0.02	0.43±0.01	0.30±0.01
	A2/5-4	0.59±0.09	0.45±0.01	0.41±0.01	0.22±0.02
	A4-1	0.49±0.02	0.45±0.01	0.44±0.02	0.56±0.09
	A4-3	0.54±0.01	0.41±0.01	0.52±0.09	0.59±0.08
	A4-2/5	0.58±0.09	0.50±0.03	0.48±0.09	0.57±0.04
	A4-4	0.59±0.03	0.53±0.01	0.37±0.04	0.43±0.01
	Average	0.52	0.48	0.42	0.34
α-1,3-glucan (A^b)	A1-1	0.49±0.02	0.51±0.01	0.34±0.04	0.25±0.03
	A1-3	0.54±0.01	0.55±0.06	0.29±0.01	0.36±0.07
	A1-4	0.59±0.06	0.52±0.01	0.45±0.01	0.35±0.04
	A3-A1	0.32±0.03	0.26±0.01	0.39±0.01	0.34±0.07
	A3-3	0.62±0.06	0.36±0.06	0.39±0.08	0.43±0.05
	A3-4	0.49±0.01	0.40±0.02	0.42±0.06	0.25±0.04
	A4-1	0.47±0.04	0.53±0.06	0.49±0.01	0.37±0.05
	A4-3	0.38±0.06	0.42±0.07	0.44±0.02	0.45±0.05
	A4-4	0.54±0.01	0.57±0.01	0.44±0.08	0.35±0.01
	Average	0.49	0.46	0.41	0.35
α-1,3-glucan (A^c)	A1-1	0.60±0.03	0.39±0.01	0.34±0.04	/
	A1-3	0.78±0.08	0.28±0.07	0.71±0.02	
	A1-4	0.46±0.09	0.53±0.01	0.49±0.08	
	A3-A1	0.23±0.03	0.29±0.01	0.87±0.04	
	A3-3	0.53±0.01	0.51±0.03	0.53±0.02	
	A3-4	0.96±0.04	0.66±0.02	0.53±0.01	
	A4-1	0.22±0.01	0.66±0.01	0.79±0.09	
	A4-3	0.39±0.02	0.70±0.01	0.60±0.01	
	A4-4	0.55±0.02	0.54±0.02	0.46±0.08	
	Average	0.52	0.51	0.59	
β-1,6-glucan (H)	H3-3	0.56±0.02	0.55±0.04	0.48±0.01	0.30±0.01
	H3-5	0.65±0.02	0.60±0.03	0.44±0.01	0.20±0.02
	H3-2	0.58±0.06	0.60±0.02	0.54±0.03	0.26±0.04
	H5-3	0.70±0.01	0.58±0.01	0.23±0.09	0.35±0.01
	H5-2	0.61±0.03	0.63±0.01	0.91±0.01	0.34±0.02
	H5-5	0.31±0.03	0.48±0.02	0.45±0.04	0.17±0.03
	H2-3	0.48±0.09	0.64±0.04	0.46±0.01	0.46±0.08

	H2-5	0.76±0.05	0.77±0.02	0.38±0.05	0.40±0.05
	H2-2	0.49±0.01	0.54±0.01	0.44±0.01	0.35±0.01
	Average	0.57	0.60	0.48	0.31
Chitin (Ch)	Ch1-1	0.22±0.04	0.54±0.01	0.36±0.01	0.21±0.02
	Ch1-3	0.24±0.06	0.55±0.02	0.67±0.03	0.13±0.02
	Ch1-2	0.60±0.04	0.72±0.02	0.88±0.02	0.31±0.03
	Ch3-1	0.58±0.02	0.73±0.03	0.76±0.02	0.39±0.01
	Ch3-3	0.48±0.02	0.62±0.02	0.47±0.02	0.40±0.02
	Ch3-2	0.99±0.04	0.72±0.08	0.43±0.02	0.33±0.05
	Ch2-1	0.93±0.01	0.14±0.02	0.94±0.04	0.47±0.01
	Ch2-3	0.99±0.07	0.34±0.03	0.49±0.02	0.47±0.06
	Ch2-2	0.59±0.02	0.54±0.01	0.34±0.07	0.35±0.01
	Average	0.62	0.54	0.59	0.34
Chitosan (Cs[a])	Cs1-1	0.51±0.05	/	0.41±0.05	/
	Cs1-3	0.33±0.08		0.62±0.03	
	Cs1-2	0.41±0.06		0.68±0.09	
	Cs3-1	0.09±0.04		0.97±0.03	
	Cs3-3	0.18±0.02		0.47±0.04	
	Cs3-2	0.50±0.05		0.53±0.01	
	Cs2-1	0.78±0.01		0.63±0.01	
	Cs2-3	0.72±0.01		0.44±0.09	
	Cs2-2	0.62±0.01		0.42±0.04	
		0.48		0.58	
Chitosan (Cs[b])	Cs1-1	0.72±0.05	/	0.64±0.05	/
	Cs1-3	0.92±0.08		0.85±0.03	
	Cs1-2	0.82±0.04		0.72±0.03	
	Cs3-1	0.40±0.06		0.80±0.09	
	Cs3-3	0.58±0.05		0.45±0.04	
	Cs3-2	0.47±0.02		0.54±0.01	
	Cs2-1	0.76±0.01		0.97±0.01	
	Cs2-3	0.98±0.01		0.57±0.04	
	Cs2-2	0.58±0.01		0.48±0.08	
		0.69		0.67	

Supplementary Table 5. ^{1}H-T$_{1\rho}$ and ^{13}C-T$_1$ relaxation times of polysaccharides in cell walls. Data is shown for the *C. neoformans* samples. The average values for each molecule in each sample are highlighted in bold. The data were measured using 1D ^{13}C relaxation experiments. The data are fit using single exponential equations: $I(t) = e^{-t/T_1}$. Error bars are standard deviations of the fit parameters.

Polysaccharide	Chemical shift	KN99		*cda1Δ2Δ3Δ*		R265		*cda1Δ2Δ3Δ*	
		^{1}H-T$_{1\rho}$ (ms)	^{13}C-T$_1$ (s)	^{1}H-T$_{1\rho}$ (ms)	^{13}C-T$_1$ (s)	^{1}H-T$_{1\rho}$ (ms)	^{13}C-T$_1$ (s)	^{1}H-T$_{1\rho}$ (ms)	^{13}C-T$_1$ (s)
α-1,3-glucan (a)	101.5	9.5±1.4	3.7±0.2	14.8±0.9	5.0±0.3	8.6±0.8	3.4±0.3	7.5±0.6	2.6±0.2
	85.0	14.2±1.0	4.1±0.1	21.7±1.4	6.2±0.5	7.4±0.7	2.7±0.2	7.6±0.6	2.5±0.2
	71.9	8.3±0.8	3.1±0.2	12.6±0.7	4.3±0.3	13.2±1.0	2.1±0.2	13.3±0.9	4.1±0.2
	Average	10.6	3.7	16.4	5.1	9.7	2.7	9.5	3.1
Chitin	104.6	7.4±0.7	1.5±0.2	3.0±0.5	1.6±0.2	4.0±0.5	1.3±0.1	5.4±0.5	1.3±0.2
	83.4	7.0±0.9	2.8±0.1	5.6±0.6	3.5±0.3	4.1±0.4	0.8±0.07	11.5±0.5	2.5±0.2
	55.9	3.8±0.4	1.6±0.1	6.2±0.4	1.6±0.2	7.6±0.8	2.1±0.2	9.2±0.6	3.1±0.1
	Average	6.0	2.0	5.0	2.2	5.3	1.4	8.7	2.3
β-1,6-glucan	103.9	5.7±0.4	1.2±0.1	7.3±0.7	2.2±0.3	3.4±0.2	0.7±0.06	6.1±0.5	1.6±0.2
	76.4	3.6±0.3	0.9±0.07	8.8±0.5	0.7±0.05	4.9±0.4	0.5±0.03	6.5±0.5	1.4±0.1
	70.6	6.5±0.7	2.5±0.2	11.4±0.5	3.1±0.4	6.2±0.5	1.4±0.2	4.0±0.3	0.6±0.06
	Average	5.3	1.5	9.2	2.0	4.8	0.8	5.5	1.2

Supplementary Table 6. ^{13}C Solid-state NMR experimental parameters for fungal cell wall characterization. T = sample temperature; B_0 = magnetic field; ν_{MAS} = MAS frequency; ns = number of scans; d_1 = recycle delay between scans; $t_{1,max}$ = maximum t_1 evolution time (for indirect dimension); $t_{1,inc}$ = increment for t_1 (for indirect dimension) evolution time; τ_{dw} = dwell time during direct FID acquisition; τ_{acq} = maximum acquisition time during direct FID detection; τ_{XY} = cross-polarization contact time during CP from channel X to channel Y; $\nu_{1H,dec}$ = dipolar decoupling field strength**.** Spin diffusion (SD).

Experiment	NMR Parameters													Samples
	T (K)	B_0 (T)	ν_{MAS} (kHz)	ns	d1 (s)	$t_{1,max}$ (ms)	$t_{1,inc}$ (µs)	τ_{dw} (µs)	τ_{acq} (ms)	τ_{HC} (ms)	τ_{SD} (ms)	τ_{mix} (ms)	$\nu_{1H\ dec}$ (kHz)	
Identification and quantification of polysaccharides														KN99, R265, *cda1Δ2Δ3Δ*
1D ^{13}C CP	298	18.8	15	1024	2			5	18	1			83	
1D ^{13}C DP	298	18.8	15	512	2 or 35			5	18				83	
2D ^{13}C-^{13}C with CORD mixing	298	18.8	15	32	2	7.5	25	5	14	1		53 τ_{CORD}	83	
2D ^{13}C-^{13}C refocused CP J-INADEQUATE	298	18.8	15	16	2	7.5	22	5	14				83	
2D ^{13}C-^{13}C refocused DP J-INADEQUATE	298	18.8	15	16	2	7.5	22	5	14				83	
PAR	280	18.8	15	32	2	7.5	20	5	20.5	1		15	83	R265, *cda1Δ2Δ3Δ*
Estimation of site-specific hydration of polysaccharides														KN99, R265, *cda1Δ2Δ3Δ*
2D ^{13}C-^{13}C water-edited	280	9.4	15	64	2	5.5	50	8	16	1	0, 4	50 τ_{PDSD}	71	
Dynamics of polysaccharides														
1D ^{13}C-T_1	298	9.4	15	512	2			8	16	1			71	
1D ^{1}H-$T_{1\rho}$	298	9.4	15	512	2			8	16	1			71	

Supplementary Reference

1 Chakraborty, A. *et al.* A molecular vision of fungal cell wall organization by functional genomics and solid-state NMR. *Nat. Commun.* **12**, 6346 (2021).

2 Lowman, D. W. *et al.* New insights into the structure of (1→ 3, 1→ 6)-β-D-glucan side chains in the Candida glabrata cell wall. *PloS one* **6**, e27614 (2011).

3 Kang, X. *et al.* Molecular architecture of fungal cell walls revealed by solid-state NMR. *Nat. Commun.* **9**, 2747 (2018).

4 Fernando, L. D. *et al.* Structural polymorphism of chitin and chitosan in fungal cell walls from solid-state NMR and principal component analysis. *Front. Mol. Biosci.* **8**, 727053 (2021).

5 Bacon, B. E., Cherniak, R., Kwon-Chung, K. J. & Jacobson, E. S. Structure of the O-deacetylated glucuronoxylomannan from *Cryptococcus neoformans* Cap70 as determined by 2D NMR spectroscopy. *Carbohydr. Res.* **283**, 95-110 (1996).

6 Hargett, A. A. *et al.* The structure of a *Cryptococcus neoformans* polysaccharide motif recognized by protective antibodies: A combined NMR and MD study. *Proc. Natl Acad. Sci. U.S.A.* **121**, e2315733121 (2024).

7 Previato, J. O. *et al.* Distribution of the O-acetyl groups and β-galactofuranose units in galactoxylomannans of the opportunistic fungus *Cryptococcus neoformans*. *Glycobiology* **27**, 582-592 (2017).

8 Ankur, A. *et al.* Polymorphic α-Glucans as Structural Scaffolds in *Cryptococcus* Cell Walls for Chitin, Capsule, and Melanin: Insights From ^{13}C and ^{1}H Solid-State NMR. *Angew. Chem. Int. Ed* **64**, e202510409 (2025).

9 Shim, J.-H. *et al.* Antitumor Effect of Soluble beta-1, 3-Glucan from *Agrobacterium* sp. R259 KCTC 1019. *J. Microbiol. Biotechnol.* **17**, 1513-1520 (2007).

10 Fairweather, J. K., Him, J. L. K., Heux, L., Driguez, H. & Bulone, V. Structural characterization by 13C-NMR spectroscopy of products synthesized *in vitro* by polysaccharide synthases using 13C-enriched glycosyl donors: application to a UDP-glucose:(1→ 3)-β-d-glucan synthase from blackberry (*Rubus fruticosus*). *Glycobiology* **14**, 775-781 (2004).

11 Saitô, H., Ohki, T. & Sasaki, T. A 13C-nuclear magnetic resonance study of polysaccharide gels. Molecular architecture in the gels consisting of fungal, branched (1→ 3)-β-D-glucans (lentinan and schizophyllan) as manifested by conformational changes induced by sodium hydroxide. *Carbohydr. Res.* **74**, 227-240 (1979).
